# The Effect of Moisture on Cellulose Nanocrystals Intended as a High Gas Barrier Coating on Flexible Packaging Materials

**DOI:** 10.3390/polym9090415

**Published:** 2017-09-05

**Authors:** Ghislain Fotie, Riccardo Rampazzo, Marco Aldo Ortenzi, Stefano Checchia, Dimitrios Fessas, Luciano Piergiovanni

**Affiliations:** 1 DeFENS—Department of Food, Environmental and Nutritional Sciences, Università degli Studi di Milano, Via Celoria 2, Milano 20133, Italy; Ghislain.Fotie@unimi.it (G.F.); Dimitrios.Fessas@unimi.it (D.F.); 2 Department of Chemistry, Università degli Studi di Milano, Via Golgi 19, Milano 20133, Italy; Riccardo.Rampazzo@unimi.it (R.R.); Marco.Ortenzi@unimi.it (M.A.O.); Stefano.Checchia@esrf.fr (S.C.); 3 CRC Laboratorio di Materiali e Polimeri (LaMPo), Department of Chemistry, Università degli Studi di Milano, Via Golgi 19, Milano 20133, Italy; 4 ESRF—The European Synchrotron, 71 Avenue des Martyrs, Grenoble 38000, France

**Keywords:** cellulose nanocrystals, flexible packaging materials, oxygen barrier, moisture effects

## Abstract

Cellulose nanocrystals (CNCs) exhibit outstanding gas barrier properties, which supports their use as a biobased and biodegradable barrier coating on flexible food packaging materials. As highly hydrophilic biopolymers, however, CNCs have a strong sensitivity to water that can be detrimental to applications with fresh foods and in moist conditions due to the loss of barrier properties. In this work, the oxygen and water vapor permeability of polyethylene terephthalate (PET) films coated with CNCs obtained from cotton linters were measured at varying levels of relative humidity, both in adsorption and desorption, and from these data, the diffusion and solubility coefficients were estimated. Therefore, the characterization of CNCs was aimed at understanding the fundamentals of the water-CNCs interaction and proposing counteractions. The CNCs’ moisture absorption and desorption isotherms at 25 °C were collected in the range of relative humidity 0–97% using different techniques and analyzed through GAB (Guggenheim-Anderson-de Boer) and Oswin models. The effects of moisture on the water status, following the freezable water index, and on the crystal structure of CNCs were investigated by Differential Scanning Calorimetry and by X-ray Powder Diffraction, respectively. These findings point to the opportunity of coupling CNCs with hydrophobic layers in order to boost their capabilities as barrier packaging materials.

## 1. Introduction

In any cellulosic material, the water content and interactions with the material’s components have a great influence on its final properties. In particular, the way in which water molecules interact with cellulose and their distribution within the often complex and heterogeneous system of cellulosic materials are critical to their applications. In fact, the manufacturing processes developed for paper, board and regenerated cellulose (cellophane films) include specific operations aimed at the careful removal of water added or present, and several hydrophobic protective layers and ingredients for providing moisture resistance are extensively used nowadays [1]. A great deal of scientific literature on this topic focuses on water sorption isotherms and other means of studying the interaction of water with cellulose, hemicellulose and lignin components, which are key components of materials in a wide range of applications [2,3,4,5,6].

Sorption isotherms interpretation, particularly through FTIR spectroscopy studies, led to the idea that water molecules are adsorbed to specific sites, both as layered adsorption or as cluster formation. The main potential adsorption sites are the hydroxyl groups and their possible oxidation forms such as the carboxyl groups [7]. For specific cellulosic materials, even at relative humidity( RH) values below 100%, it was possible to establish the number (1.0–1.3) of water molecules that adsorb to a single hydroxyl group [8,9,10]. Moreover, the evidence of two different mechanisms with slower and faster rates of water molecule exchange, suggested different sorption sites and different kinds of amorphous regions [11]. Studies of the diffusion of water molecules in cellulosic materials led to the conclusion that cellulose wetting can be related to acid-base interactions, weak hydrogen bonding and van der Waals forces (dipole-dipole and dispersion forces) [12]. Therefore, the extent of water sorption in cellulosic materials can also depend on the surface free energy of the solid-liquid interface.

At a larger scale, bulk amorphous regions have been pointed out, from as early as 1949, as a favorable place for water adsorption, and the relationship between availability of surface hydroxyl groups and crystallinity of cellulose is well established [13,14]. Nevertheless, hydrophilicity is also influenced by the supramolecular structure, the fibers network (when present), the porosity and the sponge structure exhibited by some cellulosics, namely microcrystalline cellulose (MCC). These particular morphological factors are strictly related to the manufacturing processes of the cellulosic materials, such as pulping, bleaching and refining, and they affect moisture sensitivity at different levels and in a number of ways. As a result of the heterogeneous and complex morphology of cellulosic materials, the state of water in cellulose was classified in three different types: free water (type I), freezing bound water (type II) and non-freezing bound water (type III) [15,16].

When cellulose based materials are used in packaging applications, water adsorption and desorption phenomena during the commercial life are of great importance because they are able to affect fundamental performance, such as mechanical and diffusional properties. Mechanical resistance, for instance, can be strongly reduced by moisture adsorption leading to the dissolution of the hydrogen bonds which produces tight fibers in papers and boards. This bonding occurs as the wetted fibers can dry in contact with each other, requiring close proximity between adjacent hydroxyl groups (0.25–0.35 nm) [12]. At least in paper, this bonding seems more important than the mechanical interlocking of cellulose fibers. Furthermore, the adsorption of hydrophobic components on cellulose fibers during paper manufacturing greatly decreases fiber bonding and can affect the weakness and adhesion as well [17], leading to the risk of failure and unsuitability.

The effects of water sorption on the diffusional properties of cellulose, that is gas and water vapor permeability, have been often overlooked in the past, since most cellulosic materials employed in paper and board do not require outstanding barrier properties towards gases and water. A larger use of cellulosic materials as an alternative to common synthetic polymers in food packaging has often been limited by their low performance in terms of gas and water vapor barriers.

In the recent past, such properties have been sought after, with the diffusion of regenerated cellulose films for which special coating technologies have been developed to cover and protect the cellulosic surface with lacquers based on acrylic and PVDC co-polymers [18]. Nowadays, cellulose nanocrystals and micro-nanofibrilated cellulose production has the potential [19,20] of making the moisture sensitivity and the loss of barrier properties one of the most important issues.

Several papers demonstrated the high gas barrier properties provided by the use of cellulose nanocrystals (CNCs) and cellulose nanofibers (CNFs) as coatings applied onto common plastic films, as well as fillers of common polymers [20,21,22,23,24,25]. At the same time, moisture can be seriously detrimental to the gas barrier properties of CNC coated films [19,26]. In general, synthetic or bio-based polymers with hydrophilic behavior and high polarity, show low oxygen and gas permeability when dry, but lose such properties when water molecules plasticize and swell their native structure. These behaviors are well known and widely investigated in polyamide, polyesters, polyvinyl alcohols and bio-polymers [24,26,27,28,29], while less knowledge is available for the effects of moisture on the gas barrier properties of CNCs when used as coatings applied onto conventional plastic films intended for food packaging applications, which is the main goal of this paper.

## 2. Materials and Methods

### 2.1. Materials

The cotton linters used as raw material to produce CNCs were kindly supplied by Innovhub (Milano, Italy). All the chemicals used were purchased from Sigma-Aldrich (Milano, Italy). PET film, with a thickness of 12 ± 0.5 μm, was provided by Sapici spa, Cernusco sul Naviglio, Italy.

### 2.2. CNCs Extraction by Ammonium Persulfate Treatment and Coating Process

The CNCs were obtained by the hydrolyzing-oxidative method proposed by Leung and coworkers in 2011 [30]. The procedure for the obtainment of the CNCs, the purification steps and the coating process onto PET film have been described in our previous works [19,31] and strictly followed in this paper. The yield of CNC production (%) was evaluated from the weight of the freeze-dried products by comparing them with the mass of cellulosic raw materials treated. The thickness of the coating applied onto the film was assessed by a gravimetric method. Four samples (10 × 10 cm^2^) were weighed (*m*_1_, g), then the coating was removed by running hot water (~70 °C) and the resulting uncoated film was dried and weighed (*m*_2_, g). The coating thickness (*L*, cm) was estimated by Equation (1):

*L* = (*m*_1_ − *m*_2_ )/(ρ × 100), (1)

where ρ = 1.58 g cm^−3^ is assumed as the density of the CNCs [32].

### 2.3. CNCs Morphological Characterization

By dynamic light scattering (DLS) measures (mod. Litesizer 500, Anton Paar, Graz, Austria), the equivalent hydrodynamic diameters of the CNCs were determined, as well as the polydispersity index and intensity and particle number distributions (data not shown). The measurements were performed at 25.0 ± 0.1 °C with a 35 mW laser diode light (λ = 658 nm) and collecting the scattered light at 90°. Before measurements, the samples were diluted to 1:500 (*w*/*w*) with distilled water adjusted to pH 8 and maintained at 25 °C through stirring until measurement. The diluted solutions were poured in the measurement cell after 30 s homogenization by an ultrasonic water bath. The actual dimensions of the CNCs were evaluated via Transmission Electron Microscopy (TEM). Drops of aqueous dispersions of CNC (1 wt %) were deposited on carbon-coated electron microscope grids, negatively stained with uranyl acetate and allowed to dry. Samples were analyzed with a Hitachi Jeol-10084 TEM operated (Hitachi, Brugherio, Italy ) at an accelerating voltage of 80 kV.

### 2.4. Zeta-Potential and Conductivity of the CNCs

Zeta potential (mV) and conductivity (mS cm^−1^) of the CNCs in the diluted suspension at pH 8 were determined by electrophoretic light scattering (ELS), using the PALS technology (mod. Litesizer 500, Anton Paar, Graz, Austria). Measures were replicated 5 times, at 25.0 ± 0.1 °C, by means of a 35 mW diode laser (λ = 658 nm) and at 15° detection angle.

### 2.5. X-ray Powder Diffraction (XRPD)

X-ray diffraction measurements were performed at the beamline ID15A of the ESRF synchrotron facility (Grenoble, France). The sample for X-ray diffraction was a 13 mm-diameter, 0.5 mm thick pellet obtained by pressing uniaxially 45 mg of CNC flakes. The pellet was mounted on a goniometric head and aligned normal to the incident X-ray beam at a distance of 260.5 mm from the detector, a Pilatus 2M CdTe (Dectris, Baden-Daettwil, Switzerland). Each dataset consists of 50 2D diffraction images recorded by exposing the sample for 5 s. First, the sample was measured in the dry state, i.e., as mounted. Then, 100 mg of water were pipetted on one face of the pellet and allowed to absorb immediately before collecting the second dataset. The injection of 100 mg water and the subsequent diffraction measurement were repeated 60 and 120 min after the first measurement. The X-ray wavelength was 0.17712 Å. The wavelength, sample-detector distance, detector tilt, and beam position were calibrated based on the diffraction pattern of CeO_2_. Raw diffraction images were scaled, averaged and finally subtracted by the air background before being radially integrated and corrected for the polarization of the incident X-ray beam. Calibration, image processing and radial integration were performed using Python 2.7 and the libraries pyFAI and FabIO [33,34]. For each measurement, the Pair Distribution Function (PDF) was calculated as the G(r) described by Keen, 2001 [35] using the program PDFgetX3 (Columbia Technology Ventures, New York, NY, USA) [36]. The PDF is a function in real space representing the distribution of interatomic distances in the bulk material and is obtained by Fourier-transforming the total scattering intensity after proper normalisation and correction [37]. The maximum value of momentum transfer used for PDF calculation was *Q*_max_ = 27 Å^−1^. The diffraction pattern of the dry CNC sample was fitted via the Rietveld method [38] using the program Topas 5.0 (Bruker AXS, Karlsruhe, Germany).

### 2.6. Freezable Water Content (DSC)

The freezable water content of CNCs at various humidity (*HU*% = g H_2_O/g sample) was assessed through differential scanning calorimetry (DSC) [39]. The DSC calorimeter 2920 (TA Instruments, Vimodrone, Italy), operating with 60 microliter sealed cells, was used. The typical sample mass was 30 mg; the reference cell was empty and Indium was used for calibration. Measures were carried out from −60 to 30 °C with 1.0 °C/min scanning rate. This relatively low scanning rate was selected in order to enhance maximum crystallization during cooling. Two cooling-heating cycles were performed. The transitions were reversible in the heating mode (super-cooling effects were observed in the cooling step) and the first cycle heating curves were taken into account. The output signal in mW units was divided by the product of sample mass per heating rate in order to be converted into apparent specific heat and it was scaled with respect to the baseline to obtain the excess (with respect to the water solid state) specific heat trace, *Cp*^exc^ (J K^−1^ g^−1^_water_). The heat capacity change during the solid-liquid water transition, Δ_fus_*Cp*, was scaled across the signal and was therefore not taken into account in the present work. Thanks to this treatment, the area beneath the recorded peaks directly corresponds to the relevant transition enthalpy Δ_fus_*H*. The freezable water content was assessed according the ratio Δ_fus_*H*/Δ_fus_*H*°, Δ_fus_*H*° being the pure (free) water enthalpy (333.59 J g^−1^ at 0 °C). Errors were evaluated based on at least three replicates and were under 5%.

### 2.7. Water Sorption Isotherm

The water adsorption and desorption isotherms (at 25 ± 1 °C) of CNCs were roughly determined by the standard static gravimetric method developed by the European Cooperation Project COST 90 [40], in triplicate, using saturated salt solutions to establish the RH values of 20.9%, 27.3%, 35.1%, 51.3%, 59.9%, 66.1%, 79.0% and 85.6%. In the desorption mode the Knudsen thermogravimetry approach was also used. Details of the method are reported elsewhere [39]. This method is continuous and each measurement produces the overall desorption isotherm with high reproducibility also in the low water activity ranges but can be applied only in the desorption mode [41,42,43,44]. A TG-DSC instrument (TG-DSC 111, SETARAM, Caluire, France) operating with a typical sample mass of 30 mg was used. The GAB [45,46] and Oswin [47] Equations (2) and (3) respectively, were tentatively applied to data for the adsorption and desorption of water by CNCs and shown in the following equation:

*m* = *M*o *C K aw*/[(1 − *K aw*)(1 − *K aw* + *C K aw*)], (2)

where *m* is moisture content (g H_2_O/g d.m.), *aw* is the water activity. *M*o, *K*, *C* describe the sorption properties of the structure. *M*o is the monolayer water content; the parameters *K* and *C* are the degree of freedom of water content and difference between layers (upper and monolayer) respectively and they depend on the temperature.

*m* = *c*_1_ (*aw*/(1 − *aw*)) *c*_2_, (3)

where *c*_1_ and *c*_2_ are empirical constants.

### 2.8. Optical Properties of Coated Film

The transparency of the CNCs coated PET was measured at 550 nm, according to the ASTM D 1746-70, by means of a UV-VIS spectro-photometer (mod. L650, Perkin-Elmer, Milano, Italy). The haze (%) of the same samples was measured according to ASTM D 1003-61 with the same instrument equipped with a 150 mm integrating sphere. Each sample was replicated three times, analyzing at least four spots on each replicate.

### 2.9. Water Contact Angles

Static contact angles of the coated film were determined after conditioning the samples at three different RH values (57%, 81% and 97%). The sessile drop method was used by gently dropping a droplet of 4.0 ± 0.5 μL of water onto the film. The measurements were performed at room temperature (RH about 40%) on five different positions for each sample. The equilibrium angle was achieved in 2–3 s and remained constant for at least 10–15 s; due to the short time of the measurement, we assumed that the CNCs coating did not change its original activity water value. The instrument used was an OCA 15 Plus angle goniometer (Data Physics Instruments GmbH, Filderstadt, Germany), equipped with a high-resolution CCD camera, a high-performance digitizing adapter (Data Physics Instruments GmbH, Filderstadt, Germany) and SCA20 software (Data Physics Instruments GmbH, Filderstadt, Germany) for contact angle measurements.

### 2.10. Gas and Water Vapour Permeability

All the oxygen and water vapor permeability measures were performed by an isostatic permeabilimeter (mod. Multiperm, PERMTECH S.r.l., Pieve Fosciana, Italy) according to ASTM standard methods (D-3985 and F-1249 respectively). The oxygen permeability (PO2, cm^3^ m^−2^ d^−1^ bar^−1^) of CNCs coated PET film was measured at 25 °C under 80%, 70%, 50%, 30%, 20% and 10% RH on the coated side of the film, both increasing and decreasing the RH value. The water vapor transmission rates (WVTR, g m^−2^ d^−1^) were measured at 25 °C under 90%, 80%, 70%, 60%, 50% and 40% RH on the coated side of the film (being at 0% RH on the other side). The oxygen permeability coefficients of the CNCs coating alone (KPO2, cm^3^ µm m^−2^ d^−1^ bar^−1^) were assessed using Equation (4) [48], assuming that the PET surface did not interact with the coating layer above with thickness *L* (µm), and that the interface between them negligibly affected the permeation measure.

*L*/[KPO2_(CNCs coating)_] = [1/PO2_(coated PET film)_] − [1/PO2_(uncoated PET film)_]. (4)

From the isostatic permeation curves obtained, the oxygen diffusion coefficients (*D*, cm^2^ s^−1^) in the coating at each RH value were estimated according to Equation (5) [19,49]:

*D* = *L*^2^/(7.2 × *t*_(1/2)_), (5)

where *L* is the thickness (cm) and *t*_1/2_ (s) is the time required to reach half of the maximum permeability value. From the permeability and diffusion coefficients (KPO2 and *D*), the oxygen solubility in the CNCs coating (S, bar^−1^) were estimated at each RH value, based on Equation (6):

*S* = KPO2/*D* (6)

## 3. Results

### 3.1. CNCs Production and Coating onto PET Film

**Figure 1:**
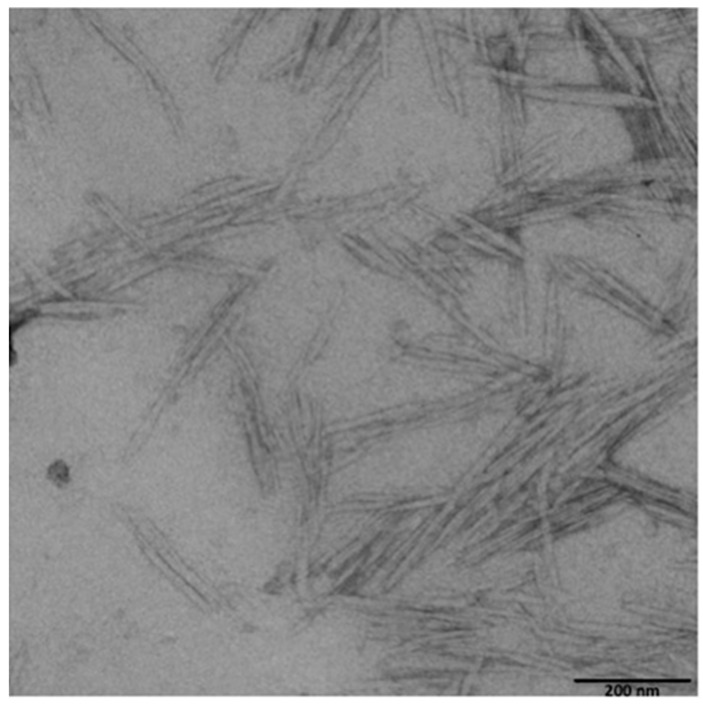
TEM micrograph of the CNCs (cellulose nanocrystals) obtained.

Cellulose nanocrystals were obtained from cotton linters by the hydrolyzing-oxidative method, already used in previous works [19,31], with a yield of about 50%; the morphological and main chemical characteristics of the CNCs were identical to the ones already described and are recalled in Table 1. As mentioned in the Materials and Methods section, the dimensions of cellulose nanocrystals were determined via transmission electron microscopy and confirmed the values obtained previously (Table 1). A TEM image of CNCs is reported in Figure 1. The coating process was also the same as described in previous papers and the tests carried out confirmed the continuity and the uniformity of the CNCs layer coated onto the PET substrate. Therefore, only a few characteristics of the coated film are reported in Table 1.

It is worth underlining that the water optical contact angles measured on coated film decreased linearly (*R*_2_ = 1.0) from 57% to 97% RH, showing a clear growth of hydrophilic behavior of the CNCs surface. Previous measures [19] on the same CNCs coated film at 35% RH, had shown a much higher value of 23.6 ± 4.9—not linearly correlated to the contact angles measured in these trials—as preliminary evidence of a dramatic change of the structure, occurring above a threshold of humidity content.

In general, the CNCs coated film obtained had an appearance and performance very similar to the previously obtained films and to many common flexible packaging materials.

### 3.2. Water and Gas Permeability of Coated PET Film

**Figure 2:**
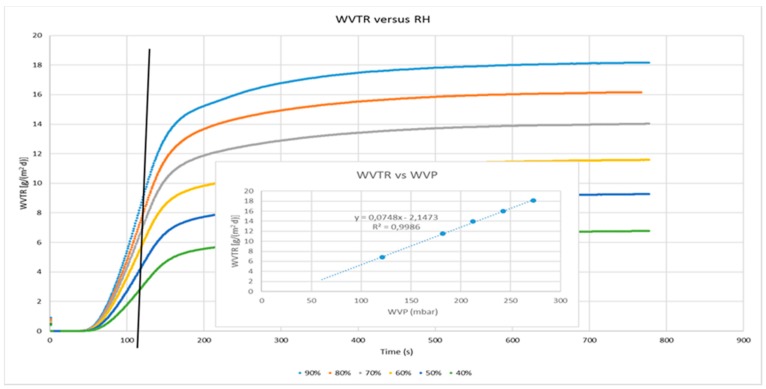
Isostatic curves of water diffusion at 25 °C under different driving forces through the CNCs coated PET film.

The water vapor transmission rates (WVTR, g m^−2^ d^−1^) were measured in duplicate at 25 °C, decreasing the RH on the coated side from 90% to 40% for each sample. The inset of Figure 2 shows the progressive and linear decrease of water transmission across the coated film, according to the water vapor pressure established by the conditions of the measures (temperature and ΔRH). Figure 2 depicts the superimposed isostatic curves of water diffusion at the different driving forces, from which it should be possible to estimate the diffusion coefficient (*D*), once *t*_1/2_ (the time required to reach half of the maximum diffusion) has been estimated. The vertical black bar shows that the *t*_1/2_ of all the curves are reached at the same time, denoting the same diffusion coefficient of water permeating through the coated film at the various driving forces considered.

**Figure 3:**
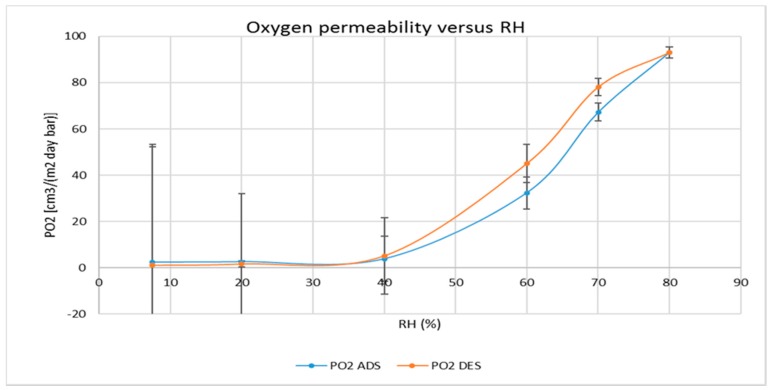
Oxygen permeability at 25 °C under different relative humidity (RH) values, both in adsorption and desorption, through the CNCs coated PET film.

The oxygen permeability of the CNCs coated PET film was also measured at various RH values in triplicate and at 25 °C, progressively changing the relative humidity on the coated side of the films from 7.7% up to 80% and then going back from 80% to 7.7% on each sample. Figure 3 reports the average values (±relative standard deviation) of oxygen permeability during the adsorption run (blue curve) and the desorption run (orange curve). Significant differences are present along the range 40−80% between the two behaviors (in adsorption and desorption) and the values increase more than 90 times from the minimum to the maximum RH value.

The isostatic permeabilimeter used has provided—also for oxygen permeability measures—sharp curves of diffusion (oxygen transmission rate versus time, data not shown) which permitted us to estimate, from the oxygen permeability coefficient of the CNCs coating layer alone (excluding the PET contribution), the diffusion (*D*) and solubility (*S*) coefficients (Equations (4)–(6)).

**Figure 4:**
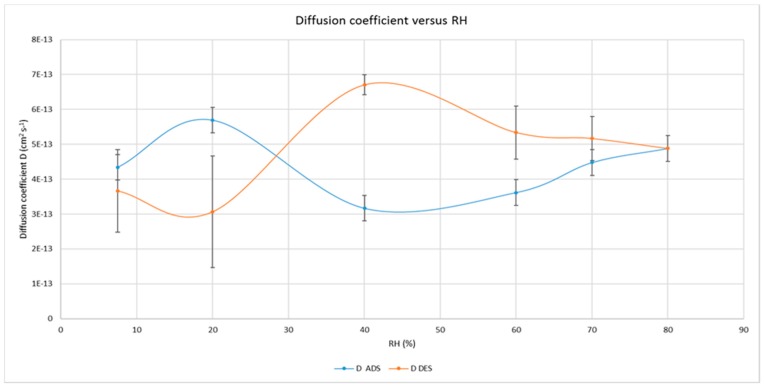
Oxygen diffusion coefficients under different RH values, both in adsorption and desorption, through the CNCs coated PET film.

**Figure 5:**
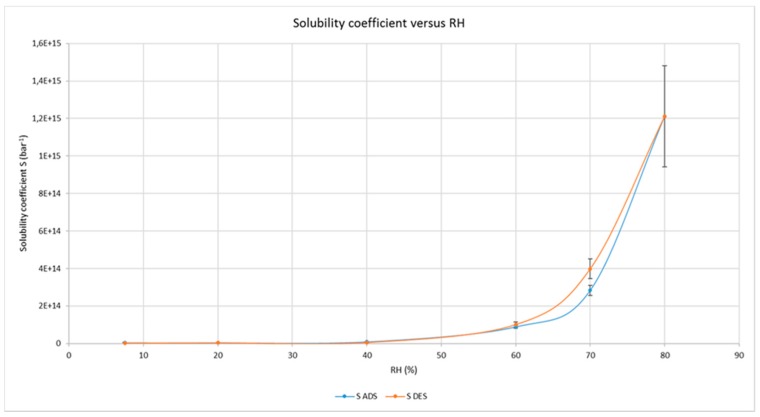
Oxygen solubility coefficients under different RH values, both in adsorption and desorption, through the CNCs coated PET film.

These fundamental parameters of the diffusional behavior of oxygen inside the cellulose nanocrystals (mean values ± standard deviation) are proposed in Figure 4 and Figure 5, as function of relative humidity values, showing large differences.

While the diffusion coefficients are quite different when measured in adsorption and desorption, with the largest and statistically significant differences between 40% and 60% RH values, the solubility coefficients remain quite constant until 70% and show much lower differences between adsorption and desorption which, however, are significant (*p* < 0.05%) at 70% RH only.

### 3.3. CNCs Freezable Water Content

**Figure 6:**
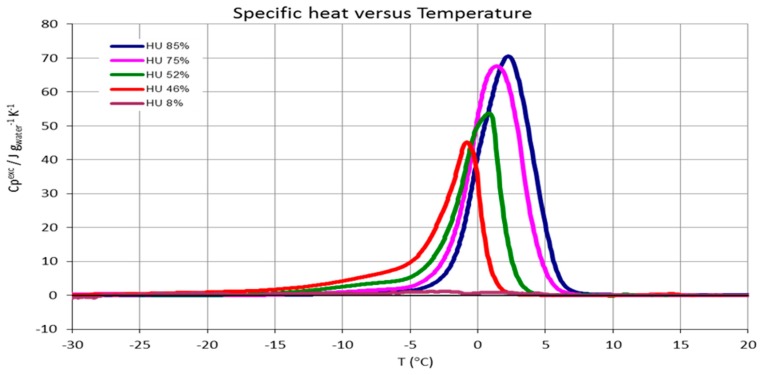
DSC measurements of CNCs at different humidity values.

In Figure 6 the DSC measurements are presented in order to assess the freezable water amount in the CNCs samples. We observe a typical behavior for water engaged in polysaccharide substrates (shift of fusion temperature due to colligative effects etc.) [50]. The profiles also indicate that the water crystal size distribution (the width of the peaks almost follow their height) is almost uniform. The freezable water results obtained following the relative enthalpies (see Materials and Methods) are reported in Table 2.

### 3.4. CNCs Water Sorption Isotherms

**Figure 7:**
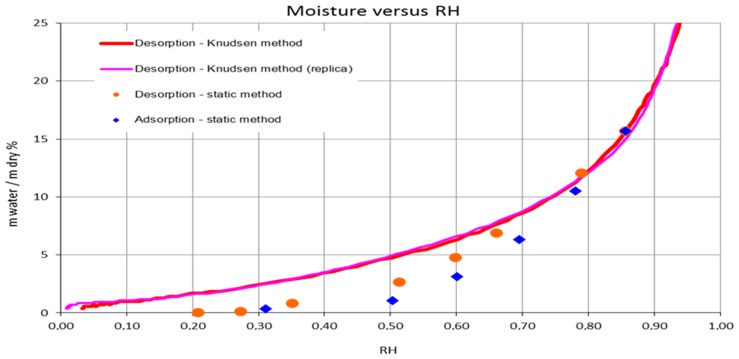
CNCs adsorption and desorption isotherm at 25 °C according to the standard static gravimetric method, and desorption isotherms according the Knudsen thermogravimetry method.

In Figure 7 we can observe the sorption isotherms of CNCs. The static gravimetric method used for roughly estimating the sorption behavior of CNCs gave satisfactory results in the middle-high region of water activity *aw* (or relative humidity RH) values, with low standard deviations. It is very likely that the values collected at lower RH were less accurate; on the other hand, the desorption isotherms obtained with the Knudsen thermogravimetry method fulfilled this gap. Indeed, we observed that these curves were highly reproducible and in agreement with the static method in the middle-high region of *aw*. In Figure 7 we also report the adsorption data obtained with the static method. Despite the uncertainties, it is interesting to highlight the hysteresis observed by the two static method curves which denotes a different behavior in adsorption and desorption steps than usually observed for similar systems. In order to be compared, the GAB equation was tentatively applied to these experimental data (excluding the points with high uncertainty, *R*_2_, respectively = 0.989, 0.810) both for the adsorption and desorption of water by CNCs. For the validation of the GAB model, *K* should be between 0 and 1, and C between 0 and 2 or higher for an isotherm of type III, as in this case. The OSWIN model also fitted well with the experimental data but is less useful for the interpretation of the water-CNCs interaction. The coefficients are reported in Table 3.

### 3.5. X-ray Diffraction Patterns

**Figure 8:**
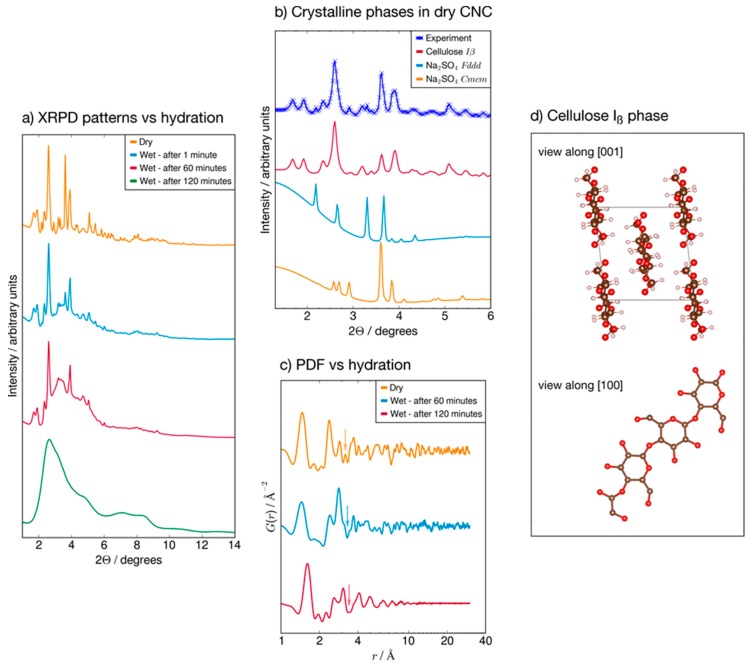
Raw XRPD (X-ray Powder Diffraction) patterns of CNCs in the dry state and after successive exposures to 100% RH (**a**), breakdown of the XRPD pattern of the dry CNCs sample into the constituent crystalline phases (**b**), the PDF curves of the dry sample and of the wet sample at the second and third hydration (**c**) and a view of the crystal structure of CNC along the crystallographic directions {001} and {100} (**d**).

In Figure 8 the panel (a) illustrates the diffraction pattern of the dry CNC sample. Panel (b) in the same figure shows the meltdown of the diffraction peaks of the CNC pellet between the four different hydration stages. Finally, the panel (c) shows the decay of the amplitude of the PDF, which directly represents the distribution of atom pairs through space in the bulk material as a function of their interatomic distance.

## 4. Discussion

### 4.1. Permeability of CNCs Coated Film

The water vapor transmission rate measures were carried out starting from 90% of RH and then decreasing the humidity on the coated side, step by step until 40%, and a very linear relationship between water transmission and driving force occurred (inset of Figure 2). It is noticeable that each previous measure did not affect the following one at a lower humidity. Thus, it is reasonable to assume that the water molecules adsorbed by the coating layer of CNCs were effectively removed by the conditioning steps applied by the instrument between measures, and that each permeability measure has been conducted in the expected conditions. Otherwise, it would not have been possible to obtain such a linear relationship.

This assumption contributes to the interpretation of the results obtained for oxygen permeability assessment at various RH values. As proved in our previous papers [19,31], at RH value close to zero, the oxygen permeability of very thin CNCs coating is much lower than the PET substrate. To get the same permeability, a PET thicker than 8.0 mm would be necessary.

The determinations, carried out in triplicate on different samples of coated films, were performed increasing at first and then decreasing the relative humidity, i.e., in an adsorption and desorption way. As expected, the oxygen permeability increased exponentially during the increase of RH values, reaching values which are no longer of interest for CNCs application as a barrier layer of flexible packaging materials for perishable food products. The most interesting and novel results deal with the measures carried out in desorption, after the achievement of 80% of RH and going back to dry conditions (7.7%). The relative standard deviations of the measures confirm that the oxygen transmission in desorption is different from that in adsorption; especially in the range between 70 and 50%. The calculation of diffusion coefficients (in Figure 4) is further confirmation of this behavior, showing the largest differences in the range 60–40% of RH whereas, below and above these limits, the uncertainties of the measure exclude statistical differences. Moreover, since the solubility of oxygen in CNCs seems slightly affected by RH values of up to 70% (in Figure 5), with negligible discrepancies between adsorption and desorption (except at 70% RH), it is reasonable to conclude that moisture has a different effect on oxygen solubility and oxygen diffusion along the cellulose nanocrystals layer.

Generally, polymer crystallization influences diffusivity more than solubility [51,52] and this is in accordance with the concepts of free-volume as well as cooperative movement of gas molecules and polymer chains [53], in which diffusion of small gas molecules through a semicrystalline polymer is viewed as the movement through the amorphous regions with an increased tortuosity given by platelet-like crystallites. Obviously, a higher thickness would increase the barrier effect even more. In bio-polymers, however, the negative correlation between crystallinity and diffusion coefficient is not always confirmed. Guinault et al. [54] showed that oxygen diffusion coefficient was accelerated upon crystallization of poly(lactic acid), while the solubility coefficient decreased, remaining constant in the amorphous phase. This phenomenon was attributed to the presence of a rigid amorphous fraction, which holds larger free volume.

The experimental evidence and the, somehow, contradictory literature analysis suggested the focus should be kept as much as possible on understanding the interactions between CNCs and water molecules.

### 4.2. Freezable Water and Water Sorption Isotherms

The above observations are in line with the DSC and the sorption data. Indeed, we observed that the amount of freezable water increases considerably with respect to the sample humidity. For instance, for sample humidity of 46%—that corresponds to about 85% in terms of water/dry matter ratio—the freezable water is 65%, i.e., 35% of water is strongly engaged in interactions with the substrate. Taking into account the sorption picture (see Figure 7) that is in an upper range of 25% in terms of water/dry matter ratio we argue that the majority of the water is strongly engaged in interactions in all the experimental RH ranges of the water vapor transmission rate measures. In this strong interaction environment, the RH variation corresponds in water/dry ratio variation sufficiently to affect the oxygen permeability. Furthermore, looking at the desorption profile, the variation of water/dry ratio is enhanced to the RH 90–40% range, while for lower RH becomes modest (flat part of the desorption curve). This is coherent with the results showed in Figure 3 (oxygen permeability vs. RH). Finally, the hysteresis effects observed (see Figure 7) are also in line with the above considerations.

The GAB model obtained has 0 < *C* < 2 which means that the isotherm of the CNCs may be of the type III and the OSWIN model actually fitted sharply the relationship between the moisture content and the water activity, sustaining the experimental results.

### 4.3. Cristallinity by WAXD Analyses

X-ray diffraction measurements explained the hydration behaviour of CNCs at the crystal structure level at 100% RH and, particularly, the changes in short-range and long-range interactions that make the cellulose nanocrystals (whiskers) accommodate a large amount of absorbed water.

The diffraction pattern of the dry CNCs sample shows a highly crystalline phase corresponding to the Iβ polymorph of cellulose (space group *P*112_1_), which consists of chains of pyranosidic rings directed along the c-axis (panel (d) in Figure 8), and in which the sharp reflections (012) and (102) point to the random orientation of the crystallites in the sample [55]. In addition to the cellulose phase, the dry CNCs sample contains 22% wt of Na_2_SO_4_, as evidenced by the diffraction pattern showing two different Na_2_SO_4_ polymorphs with space groups *Cmcm* and *Fddd* [56,57]. Panel (a) in Figure 8 illustrates the contribution of each of the three phases to the experimental powder pattern of the dry CNCs sample.

Panel (b) in the same figure shows the meltdown of the diffraction peaks of the CNCs pellet between the four different hydration stages, each of them exposed the CNCs pellet to 100% RH by absorption of 100 mg of water. Immediately after the first hydration (blue line, second from top), the sharp Bragg peaks of the Iβ phase are retained, while a higher background around scattering angle 2θ = 4° signals the partial amorphisation of cellulose; predictably, embedded Na_2_SO_4_ was dispersed upon contact with the absorbed water, as evidenced by the drop in the intensity of the peaks at 2θ ~ 2.20°, 3.60°. Upon swelling the pellet again by absorbing another 100 mg of water after 60 min (red curve, third from top), the amorphous regions in the sample expanded at the expense of the crystalline part; the most intense Bragg peaks of cellulose are still visible but the large amorphous bump now dominates the diffraction pattern. After the third and last exposure to water (green curve, bottom), all the Bragg peaks vanished and the diffraction pattern only showed a broad, amorphous feature.

The loss of structural coherence can be evidenced even more clearly by the decay of the amplitude of the PDF (panel (c) in Figure 8), which directly represents the distribution of atom pairs through space in the bulk material as a function of their interatomic distance.

While the PDF curves relative to the dry sample (orange line, top) and after 60 min (blue line, middle) have discernible peaks up to *r* = 40 Å, the PDF collected after 120 min (red line, bottom) is flat for every interatomic distance above *r* = 12 Å. The smearing of the interatomic distances at the nanometer scale is indicative of the broad distribution of inter-chain distances in the cellulose crystallites brought about by hydration. At the same time, several intra-chain interatomic distances within 10 Å are preserved but show crucial changes that are directly relatable to structural changes in CNCs upon hydration. At the second hydration, after 60 min (blue line, middle), the peak at *r* = 2.50 Å becomes broader and weaker than in the dry sample: besides the disappearance of the underlying Na–O distances as Na_2_SO_4_ is dispersed in water, this suggests a broader distribution of the distances between neighbouring pyranosidic rings; the sharp peak at 2.87 Å, on the other hand, is specific to intra-ring interatomic distances and changes only slightly with respect to dry CNCs. The disappearance of the peak at *r* = 3.17 Å suggests that the benzyl alcohol moieties engage in different H-interactions and they no longer sit in the same plane as the C-alpha (see the {100} projection in panel (d)). The third hydration, after 120 min, results in the general broadening of the PDF features which broaden even within *r* = 10 Å; beyond this distance, corresponding to two pyranosidic rings, the PDF is then completely flat and corresponds to the complete loss of structural coherence.

## 5. Conclusions

The multidisciplinary approach to understanding the water-cellulose nanocrystals interaction resulted in a complex scenario, leading to a unique conclusion about the importance of preserving the gas barrier properties exhibited by CNCs, by limiting as much as possible the moisture adsorption. In the case of practical applications such as food-packaging materials, the presence of a hydrophobic and sealable polymeric layer protecting the CNCs coating seems essential. However, an identical issue concerns synthetic polymers, like polyvinyl alcohol (PVOH) or ethylene vinyl alcohol copolymer (EVOH), which are even less of a barrier than CNCs.

We observed that at low humidity the amount of freezable water decreases considerably indicating relevant interaction with the substrate, despite the accessible surface area being limited by the crystallinity of cellulose nanoparticles. The freezable water, however, seems not strictly related to the oxygen barrier, since we measured very low permeability values up to about 40% RH, where the freezable appears higher than 50%.

The water adsorption by CNCs is relevant and continues to follow an isotherm of type III. This phenomenon provokes the loss of structural coherence, as clearly evidenced by the X-ray diffraction patterns, and the sharp increase of oxygen diffusion and solubility, leading to permeability values of no interest for packaging applications.

## Figures and Tables

**Table 1:** Main characteristics of cellulose nanocrystals and of the CNCs coated PET film.

Property of CNCs	Value ^1^
Hydrodynamic diameter (nm)	101.15 ± 3.65
Average dimensions (length, *L*) from TEM measures	139 ± 33
Average dimensions (diameter, *D*) from TEM measures	16 ± 5
Aspect ratio (*L*/*D*)	9 ± 4
Zeta potential (mV)	−44.40 ± 4.12
Conductivity (mS cm^−1^)	0.095 ± 0.024
Polydispersity index	22.95 ± 0.63
*aw* after freeze drying	0.26 ± 0.01
Property of CNCs coated PET film	
*aw* after coating and drying	0.46 ± 0.05
Thickness of PET film (µm)	12.0 ± 1
Thickness of CNCs coating (nm)	756.3 ± 22.3
Transparency (T% at 550 nm)	85.67 ± 0.3
Haze (%)	1.89 ± 0.1
Optical contact angle (water) at 57% RH	11.23 ± 0.41
Optical contact angle (water) at 81% RH	9.33 ± 0.56
Optical contact angle (water) at 97% RH	8.05 ± 0.31

^1^ Mean values of at least 3 replicates ± standard deviations.

**Table 2:** CNCs freezable water content assessed by DSC.

CNCs humidity % (g H_2_O/g sample)·100	Freezable water % ± 5%
85	100
75	100
52	75
46	65
8	0

**Table 3:** Empirical coefficients of GAB and OSWIN models fitting the experimental isotherms.

Coefficient	Adsorption	Desorption
	GAB equation	
*R*_2_	0.989	0.810
*k*	1.27	1.95
*C*	0.14	0.35
*M*o	0.45	0.08
	OSWIN equation	
*c*_1_	1.3	1.23
*c*_2_	1.57	1.90
*R*_2_	0.97	0.90
